# Effect of total number of harvested lymph nodes on survival outcomes after curative resection for gastric adenocarcinoma: findings from an eastern high-volume gastric cancer center

**DOI:** 10.1186/s12885-017-3872-6

**Published:** 2018-01-12

**Authors:** Shiela S. Macalindong, Kwang Hee Kim, Byung-Ho Nam, Keun Won Ryu, Norihito Kubo, Ja Yeon Kim, Bang Wool Eom, Hong Man Yoon, Myeong-Cherl Kook, Il Ju Choi, Young Woo Kim

**Affiliations:** 1 Gastric Cancer Branch, Research Institute and Hospital, National Cancer Center, Goyang, Republic of Korea; 2 Department of Surgery, Philippine General Hospital, University of the Philippines, Manila, Philippines; 3 Biometric Research Branch, National Cancer Center, Goyang, Republic of Korea; 4 Department of Gastroenterological Surgery, Hirosaki University Graduate School of Medicine, Aomori, Japan

**Keywords:** Gastric cancer, Lymph node harvest/retrieval, Survival

## Abstract

**Background:**

Greater lymph node retrieval in gastric cancer improves staging accuracy and may improve survival from increased clearance of nodal micrometastasis. This retrospective cohort study investigated if more lymph nodes removed in gastric cancer increases survival and if such effect is stage-specific due to differential risks of nodal micrometastasis and systemic disease.

**Methods:**

The prospectively collected database of curatively resected gastric cancer patients in National Cancer Center, South Korea between 2000 and 2009 was reviewed. Disease-free survival (DFS) and overall survival (OS) for all patients and for each stage according to number of lymph nodes examined (1–30, 31–45, > 45) were analyzed.

**Results:**

Of 4049 patients, 96.6% and 98.4% underwent D2 (perigastric and extragastric) lymphadenectomy and had ≥ 15 lymph nodes examined. Mean number of nodes examined was 43. Five-year OS & DFS rates were 83.3% and 80.7%. Patients with > 45 nodes examined had significantly lower DFS (*p = 0.002*) and OS (*p = 0.007*) compared to those with 1–30 and 31–45 nodes. However, proportion of patients with > 45 nodes examined increased with stage (*p = 0.0005*). Per stage, there was no significant difference in DFS and OS according to number of nodes examined except for stage IIIA favoring more nodes (*p = 0.018* and *p = 0.044,* respectively). Similar trend was seen in stage IIB. Number of examined nodes positively correlated with number of pathologic nodes for all patients (*r* = 0.144, *p* < .001) but not for stage IIB and IIIA. Number of nodes examined was a significant survival predictor in stage IIIA.

**Conclusion:**

Greater lymph node harvest showed improved survival in intermediate-stage gastric cancer.

**Electronic supplementary material:**

The online version of this article (10.1186/s12885-017-3872-6) contains supplementary material, which is available to authorized users.

## Background

Gastric cancer is the 5^th^ and 3^rd^ most common cancer and cause of cancer-related mortality, respectively, worldwide [1]. Surgery, which includes appropriate gastrectomy and lymphadenectomy, is the cornerstone of treatment. Lymphadenectomy may be limited to perigastric lymph nodes (D1) or extended to include nodes along the named vessels of the celiac axis (D2). Arguments favoring extended lymphadenectomy include improved staging and locoregional control and potential survival benefit based on non-randomized reports [2–5]. While initial results from Western randomized trials failed to validate survival advantage with extended dissection [6, 7], long-term follow-up showed decreased gastric cancer-related deaths particularly in patients with limited nodal disease and without pancreaticosplenectomy [8]. This finding along with results from Eastern randomized trials showing better survival with extended lymphadenectomy [9], the non-requirement of distal pancreaticosplenectomy for all D2 dissection [10, 11], and the decreased perioperative mortality in high volume centers [12], have led to the recommendation by major consensus groups of D2 dissection as standard lymphadenectomy for gastric cancer [13–16].

Greater extent of lymphadenectomy is associated with increased number of harvested lymph nodes [17–20]. In the lymphadenectomy trials, consistently more nodes were retrieved in the D2 arms [6, 7, 9]. Several reports including population-based and single, low to high volume institution studies demonstrated better survival with increased number of removed nodes [5, 21–25]. However, it is difficult to ascertain if this effect on survival is due to stage migration or an actual therapeutic benefit [2, 3, 5, 24–26]. With more nodes examined, the probability of detecting pathologic nodes increases, leading to more accurate staging and stage-specific survival estimates [2, 20, 27, 28]. Stage migration is of particular concern in Western studies with high proportion of cases not meeting the minimum of 15 nodes examined recommended for accurate staging [5, 19, 21, 29]. Alternatively, removal of more lymph nodes may improve survival by improving locoregional control via clearance of nodes harboring macro- and micrometastasis. Nodal micrometastases in gastric cancer were shown to negatively impact survival [30–33].

We hypothesize that the therapeutic benefit of removing more lymph nodes is limited to intermediate-stage disease. In early cancer, removal of more nodes may not improve survival because nodal metastasis risk is low, while in advanced stages, risk of systemic disease is high thus offsetting any benefit achieved with improved regional control. We aim to define the impact of the number of lymph nodes removed on survival in a high-volume gastric cancer center where lymph node harvest for accurate staging is achievable and treatments standardized. The study considers whether, beyond the influence of number of lymph nodes removed on the staging quality, the removal of more nodes in itself has a therapeutic benefit and if such benefit is stage-specific.

## Methods

A review was conducted of prospectively collected data relating to histologically proven gastric adenocarcinoma patients who underwent primary curative resection in National Cancer Center (NCC), South Korea between 2000 and 2009. Nearly half of the NCC patients, both public and private, were from the regional area and the other half from a nationwide distribution. Included in the study were patients ≥18 years who underwent R0 resection with lymphadenectomy and at least 5 years follow-up for survivors. Patients with distant metastases (including peritoneum, cytology-positive, paraaortic lymph nodes], concurrent or history of other malignancy, previous gastrectomy, prior neoadjuvant chemotherapy/chemoradiotherapy, who underwent sentinel lymph node biopsy, or died within 30 days from surgery were excluded. Neoadjuvant therapy patients were excluded as neoadjuvant therapy may decrease nodal yield. Postoperative deaths were excluded since the outcome of interest is long-term survival. High-volume gastric surgeons performed the operations. A dedicated team of gastric pathologists performed the examination of lymph nodes using standardized conventional protocol.

Clinicopathologic and survival data were retrieved primarily from the NCC Center of Gastric Cancer database. Additional electronic medical records were reviewed for some patients to supply missing data. Clinicopathologic data were retrieved relating to age, sex, American Society of Anesthesiology (ASA) score, body mass index (BMI), pathologic tumor (pT) category, pathologic tumor size, mean number of positive pathologic lymph nodes, pathologic nodal status (pN) category, total number of lymph nodes examined, pathologic stage, tumor location (proximal, middle, distal, whole), histologic grade, Borrmann type, Lauren type, lymphovascular invasion (LVI), perineural invasion (PNI), resection type (subtotal, total, extended), lymph node dissection extent (D1, D2, > D2), and adjuvant chemotherapy. Patients were grouped according to pathologic stage (American Joint Committee on Cancer 7th edition) and categories defined by the total number of lymph nodes examined. Categories of number of lymph nodes examined were determined a priori and to avoid small numbers per category, particularly with few lymph nodes examined, the first group consisted of patients with 30 or less nodes, the second with 31–45 nodes and the third with more than 45 nodes. Data were presented as means (with standard deviations, SD) for continuous variables and frequencies (%, count and denominators) for categorical variables.

With the main objective of the study to investigate an association between the total number of LN examined and survival, Kaplan-Meier method was used to estimate survival curves for all stages and for each stage according to total number of lymph nodes examined. Survival was defined in terms of disease-free survival (DFS) and overall survival (OS). Log-rank test was used to detect survival differences. Cases were censored on their last known follow-up check up or with the occurrence of outcome of interest (recurrence or death from any cause). Analysis of variance (ANOVA) was used to detect differences in the total lymph nodes examined among stages. Linear regression modeling was used to test the trend with those more than 45 lymph nodes examined according to stage. Scatterplot and linear regression analyses were used to assess stage migration with number of examined lymph nodes as independent variable and number of positive pathologic lymph nodes as dependent variable. To provide additional evidence for association of total number of examined lymph with survival, univariate and multivariate analyses using the Cox proportional hazards model of this variable along with other clinicopathologic variables deemed to affect survival in gastric cancer based on previous reports (age, sex, BMI, ASA score, pT, pN, pstage, tumor location, histologic grade, Borrmann type, Lauren type, LVI, PNI, resection type and adjuvant chemotherapy) were done. Variables which showed significance (*p* value < 0.05) on univariate analysis were entered into the multivariate analysis to identify which variables remained significantly associated with survival. The analyses were not intended to develop a prognostic risk prediction model but to look at association of factors separately.

SAS version 9.3 (SAS institute Inc., Cary, NC, USA) was used in the statistical analyses and graphs were generated using R statistical software (Version 3.1.2; R Foundation for Statistical Computing, Vienna, Austria). Analyses were 2-sided and level of statistical significance was set at *p* values ≤ 0.05.

The NCC Institutional Review Board approved the study (NCC 2015–0084).

## Results

### Patient characteristics and surgical outcomes

**Fig. 1:**
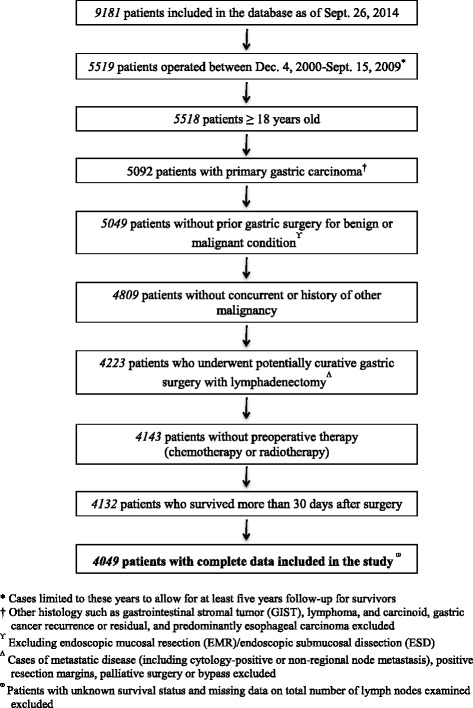
Process of case selection from the database for study inclusion

After applying the stated inclusion and exclusion criteria, 4049 patients were identified for inclusion in the study (Fig. 1). The clinicopathologic characteristics of 4049 patients are shown in Table 1. Complete data were available for 100% of cases for nine of 19 variables for which data were collected, 99% of cases for six variables, 97% for two variables, and 91% and 80% for one variable each. Median follow-up were 84.7 months (range, 1.1 to165.2) for all patients and 93.3 months (range, 46.7 to 165.2) for survivors. Nine hundred eighty-eight patients (24.4%) died within 5 years from surgery and the rest (3061 patients, 75.6%) were known to be alive more than 5 years from date of surgery. Mean number of examined lymph nodes was 42.9 (SD 15.9) while mean number of pathologically positive lymph nodes was 2.8 (SD 6.3). Ninety-eight percent (98%) of patients had more than 15 lymph nodes examined. At least a D2 dissection was performed in 96.6% of patients. During the study period, there were only 11 patients who died within 30 days postoperatively, representing 0.27% of all cases and distributed as follows according to total number of lymph nodes examined: 0–30 – 2 (0.22%), 31–45 – 5 (0.31%), and > 45–4 (0.26%). Ninety-three patients (2.25% of all cases) were excluded from the study due to receipt of neoadjuvant chemotherapy and were equally distributed across categories of total number of lymph nodes examined (0–30: 27 cases, 2.9%; 31–45: 33 cases, 2.02%; > 45: 33 cases, 2.07%).

**Table 1:** Clinicopathologic characteristics of patients

Clinicopathologic variable	Mean (SD)/ Frequency (%) *n* = 4049
Age, in years	
Mean, SD	57.6 (11.9)
< 65 years	2722 (67.2%)
≥ 65 years	1327 (32.8%)
Sex	
Male	2694 (66.5%)
Female	1355 (33.5%)
BMI, in kg/m^2^	23.7 (3.1)
ASA	
I	1540 (38.0%)
I	1991 (49.2%)
III	187 (4.6%)
Unknown	331 (8.2%)
pT size, in cm	4.6 (2.8)
pT category	
T1	2049 (50.6%)
T2	595 (14.7%)
T3	787 (19.4%)
T4	618 (15.3%)
pN category	
N0	2465 (60.9%)
N1	550 (13.6%)
N2	440 (10.9%)
N3	594 (14.7%)
Total number of pathologically positive lymph nodes	2.8 (6.3)
Total number of lymph nodes examined	
Mean, SD	42.9 (15.9)
0–30	889 (22.0%)
31–45	1597 (39.4%)
> 45	1563 (38.6%)
pStage	
IA	1815 (44.8%)
IB	501 (12.4%)
IIA	423 (10.4%)
IIB	330 (8.2%)
IIIA	303 (7.5%)
IIIB	341 (8.4%)
IIIC	336 (8.3%)
Tumor location	
Proximal	683 (16.9%)
Middle	1370 (33.8%)
Distal	1916 (47.3%)
Whole	80 (2.0%)
Histologic grade^a^	
Differentiated	1542 (38.1%)
Undifferentiated	2456 (60.7%)
Others	49 (1.2%)
Unknown	2 (0.05%)
Borrmann type	
0	2214 (54.7%)
I	51 (1.3%)
II	374 (9.2%)
III	1269 (31.3%)
IV	121 (3.0%)
V	19 (0.5%)
Unknown	1 (0.02%)
Lauren type	
Intestinal	1899 (46.9%)
Diffuse	1644 (40.6%)
Mixed	359 (8.9%)
Indeterminate/Unknown	147 (3.6%)
Lymphovascular invasion	
Present	1596 (39.4%)
Absent	2340 (57.8%)
Unknown	113 (2.8%)
Perineural invasion	
Present	948 (23.4%)
Absent	2293 (56.6%)
Unknown	808 (20.0%)
Resection type	
Subtotal	2892 (71.4%)
Total	988 (24.4%)
Extended	169 (4.2%)
Extent of lymph node dissection	
D1	126 (3.1%)
D2	3867 (95.5%)
> D2	46 (1.1%)
Unknown	10 (0.2%)
Adjuvant chemotherapy^b^	
Yes	1002 (24.7%)
No	3012 (74.4%)
Unknown	35 (0.9%)

*Abbreviations*: *BMI* body mass index, *ASA* American Society of Anesthesiology, *pT* pathologic tumor, *pN* pathologic nodal status

^a^Differentiated histology included papillary adenocarcinoma, well-differentiated tubular adenocarcinoma, and moderately differentiated adenocarcinoma. Undifferentiated histology included poorly differentiated adenocarcinoma, signet ring cell adenocarcinoma, and mucinous carcinoma. Others included adenosquamous, squamous, neuroendocrine, etc

^b^Adjuvant chemotherapy was indicated in patients with Stage II disease and higher. Adjuvant chemotherapy after publication of S1 adjuvant therapy trial and initiation and subsequent publication of adjuvant capecitabine + oxaliplatin (XELOX) trial were either of the two. Prior to these trials, 5-fluorouracil-based chemotherapy was used

Table 2 shows the number of total lymph nodes examined per stage category. By ANOVA, mean number of lymph nodes examined was significantly different among stage subgroups. Further analysis by linear regression revealed that with increasing stage, the proportion of patients with more than 45 nodes examined increased (*t* = 7.83, *p* = 0.0005).

**Table 2:** Frequency distribution of total lymph nodes examined by stage

Stage	n	Total No. of Lymph Nodes Examined^*^ Mean (SD)	Total Lymph Nodes Examined Category Frequency (%)		
			0–30	31–45	> 45†
All	4049	42.9 (15.9)	889	1597	1563
			(22.0%)	(39.4%)	(38.6%)
IA	1815	40.2 (14.8)	473	765	577
			(26.1%)	(42.1%)	(31.8%)
IB	501	41.5 (14.8)	117	207	177
			(23.4%)	(41.3%)	(35.3%)
IIA	423	45.3 (17.3)	82	162	179
			(19.4%)	(38.3%)	(42.3%)
IIB	330	44.7 (15.5)	59	120	151
			(17.9%)	(36.4%)	(45.8%)
IIIA	303	45.8 (16.8)	56	110	137
			(18.5%)	(36.3%)	(45.2%)
IIIB	341	46.7 (16.9)	60	120	161
			(17.6%)	(35.2%)	(47.2%)
IIIC	336	48.5 (16.2)	42	113	181
			(12.5%)	(33.6%)	(53.9%)

*ANOVA test: F = 24.31, *p* < 0.001

†Linear regression: t = 7.83, *p* = 0.001

### Survival outcomes by lymph node harvest groups

**Fig. 2:**
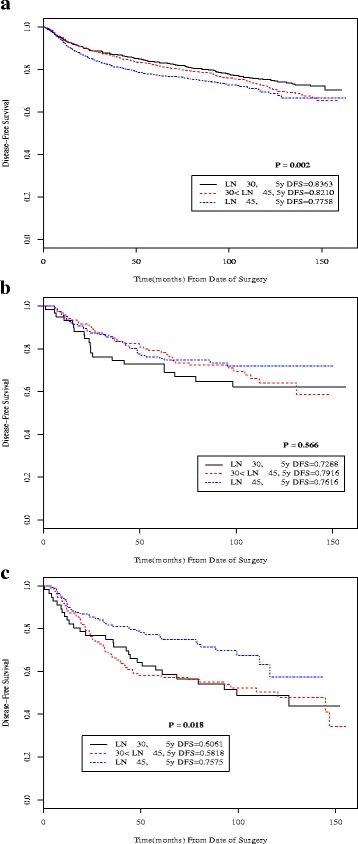
Kaplan-Meier disease-free survival curves according to total number of lymph nodes examined (≤ 30, 31–45, > 45) for all patients (**a**) and for stage subgroups IIB (**b**) and IIIA (**c**)

Five-year OS and DFS rates for all patients were 83.3% and 80.7%, respectively. Survival estimates for all patients and for each stage according to number of lymph nodes examined were obtained. (Additional files 1 and 2: Figures S1 and S2). For all patients, statistically significant differences in DFS according to total number of lymph nodes examined were seen (Fig. 2). Patients with more than 45 nodes examined had significantly lower DFS compared to patients who had 1–30 and 31–45 nodes. However, as shown in Fig. 2, when survival was analyzed by stage subgroup, stage IIIA patients showed significantly improved survival with more nodes examined (*p* = 0.018). A non-statistically significant trend for improved survival with greater nodal harvest for stage IIB was likewise observed (*p* = 0.566). For all other stages, the DFS curves for the three total lymph nodes examined categories overlapped extensively and no statistical difference was found (Additional file 1: Figure S1).

**Fig. 3:**
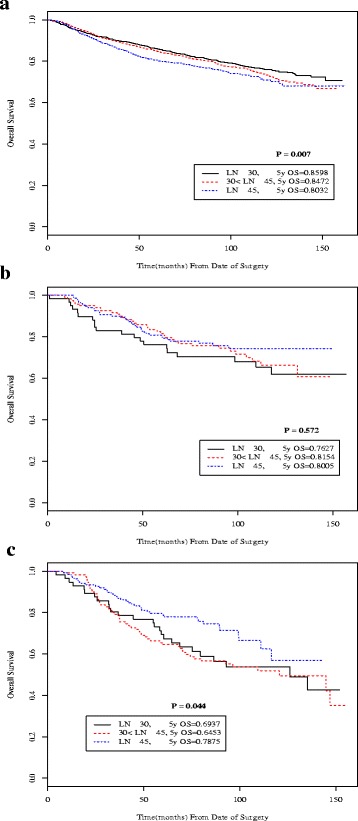
Kaplan-Meier overall survival curves according to total number of lymph nodes examined (≤ 30, 31–45, > 45) for all patients (**a**) and for stage subgroups IIB (**b**) and IIIA (**c**)

Similar results were found when comparing OS according to number of lymph nodes examined (Fig. 3). For all patients, OS was significantly different across the three categories of total lymph nodes examined, with worse outcomes seen with > 45 nodes group. Analyzed by stage subgroup, only stage IIIA patients had significantly different OS depending on the total lymph nodes examined, again favoring more nodes (*p* = 0.044). Similar trend for improved survival with greater number of total lymph nodes examined was also seen in stage IIB (*p* = 0.572).

### Stage migration effect analysis

**Fig. 4:**
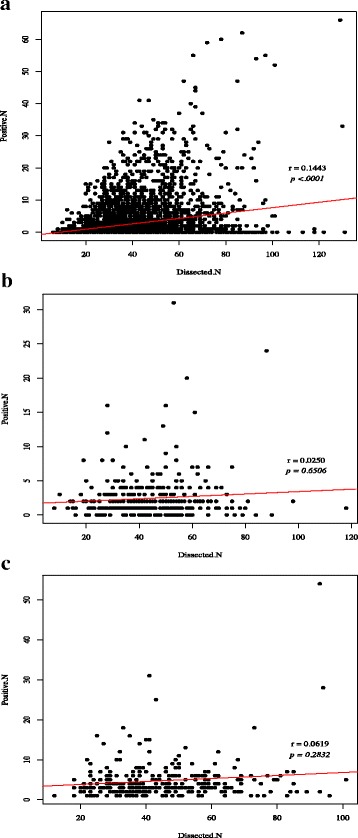
Scatterplot and linear regression analysis of number of positive pathologic lymph nodes versus number of harvested lymph nodes for all patients (**a**) and for stage subgroup IIB (**b**) and stage IIIA (**c**) (*r* Spearman correlation value*; p* Spearman correlation test value)

The scatterplot and linear regression analyses (Fig. 4) showed significant positive correlation between number of nodes examined and number of positive pathologic nodes (*r* = 0.144, *p* < .001). However, on analysis by stage subgroupings (Additional file 3: Figure S3), no significant correlation was observed in stage IIB and IIIA (*p* = 0.651 and *p* = 0.283) (Fig. 3).

### Factors affecting overall survival

In univariate analysis, all variables examined except Lauren type were significant predictors of overall survival in all patients (Additional file 4: Table S1). But, multivariate analysis identified only age, body mass index (BMI), American Society of Anesthesiology (ASA) score, pT (pT4 only) and pN category, tumor location (distal and whole only), histologic grade, Borrmann type (III and IV only), lymphovascular invasion and resection type (extended resection) as significant survival predictors (Table 3). Total number of lymph nodes examined was not a significant predictor in this analysis. For stage IIIA specifically, univariate analysis revealed age, BMI, total number of lymph nodes examined, and tumor location as significant predictors for survival (Additional file 4: Table S1). Age and total number of lymph nodes examined (> 45) remained significant in multivariate analysis (Table 3).

**Table 3:** Multivariate analysis of clinicopathologic factors associated with overall survival^†^

Risk factors	All Stages			Stage IIIA		
	HR	95% CI	*p* ^a^	HR	95% CI	*p* ^a^
Age, years						
< 65	1.00	Reference		1.00	Reference	
≥ 65	1.99	1.70–2.33	< .001	2.23	1.51–3.30	< .001
BMI	0.95	0.92–0.97	< .001			
ASA						
I	1.00	Reference				
II	1.25	1.06–1.48	0.008			
III	2.24	1.71–2.93	< .001			
pT category						
T1	1.00	Reference				
T2	0.83	0.56–1.24	0.366			
T3	1.10	0.72–1.66	0.663			
T4	2.00	1.29–3.09	0.002			
pN category						
N0	1.00	Reference				
N1	1.44	1.10–1.87	0.007			
N2	1.79	1.36–2.35	< .001			
N3	3.45	2.64–4.50	< .001			
Total number of LN examined						
0–30	*1.00*	Reference		*1.00*	*Reference*	
30–45	*1.05*	*0.86–1.28*	*0.614*	*0.87*	*0.53–1.45*	*0.580*
> 45	*0.96*	*0.79–1.18*	*0.721*	*0.56*	*0.33–0.95*	*0.030*
Tumor location						
Proximal	1.00	Reference				
Middle	1.27	1.00–1.61	0.051			
Distal	1.35	1.05–1.75	0.020			
Whole	1.66	1.15–2.40	0.007			
Histologic grade*						
Differentiated	1.00	Reference				
Undifferentiated	0.85	0.72–1.00	0.045			
Others	2.69	1.74–4.15	< .001			
Borrmann type						
0	1.00	Reference				
I	1.73	0.92–3.25	0.089			
II	1.43	0.95–2.15	0.085			
III	1.85	1.28–2.68	0.001			
IV	2.27	1.41–3.65	0.001			
V	1.29	0.31–5.42	0.732			
Lymphovascular invasion						
Absent	1.00	Reference				
Present	1.31	1.08–1.60	0.007			
Resection type						
Subtotal	1.00	Reference				
Total	1.20	0.98–1.49	0.084			
Extended	1.55	1.16–2.06	0.003			

*Abbreviations*: *BMI* body mass index, *ASA* American Society of Anesthesiology, *pT* pathologic tumor, *pN* pathologic nodal status

^†^Only factors found to be statistically significant, except for total number of LN examined, reported. For all variables, refer to Additional file 4: Table S1

^a^*p* values indicate a relative statistical significance for each category compared to the reference category in each variable (HR 1.00)

*Differentiated histology included papillary adenocarcinoma, well-differentiated and moderately-differentiated tubular adenocarcinoma. Undifferentiated histology included poorly differentiated adenocarcinoma, signet ring cell adenocarcinoma, and mucinous carcinoma. Others included adenosquamous, squamous, neuroendocrine, etc.

## Discussion

The study results suggest a therapeutic benefit with removal of more lymph nodes that is limited to patients with intermediate-stage disease. In early disease, harvesting more nodes may not have survival gains because the risk of nodal metastases is low at the outset [3]. Also, nodes with both macro and micrometastases are limited and may not necessitate large number of nodes to be removed for complete clearance. In advanced stages, the risk of disease already being systemic is high so that clearance of more nodes potentially harboring micrometastasis is of minor therapeutic consequence. In intermediate-stage disease, however, harvesting of more lymph nodes with potential micrometastasis in a disease setting that is likely still locoregional may have a therapeutic impact. This may account for the statistically significant association with improved survival for stage IIIA disease and a favorable trend in stage IIB.

That survival benefit associated with the removal of more lymph nodes is limited to a particular intermediate-stage subgroup in this study is in line with other reports. In the Dutch trial, N2 subgroup had better survival with D2 dissection [8]. Karpeh et al. showed that examination of 15 or more nodes significantly influenced survival estimates for stage II and III gastric cancer [25]. In a prospective multicenter observational study, extent of lymphadenectomy, defined according to number of nodes removed (≤ 25 versus > 25), had an independent survival effect in stage II disease [34]. This effect persisted even after exclusion of patients with insufficient nodal dissection [34]. A South Korean high-volume center retrospective study showed that in patients with >15 nodes examined, there was no stage-specific survival differences according to nodal yield except for stage IIIB, an effect attributed to improved regional control with removal of lymph nodes with microinvolvement [35]. The lack of survival advantage with higher lymph node yield in early gastric cancer in this study is consistent with other reports [24, 35] and existing recommendations for limited lymphadenectomy (D1 or D1+) for early (cT1N0) gastric cancer [15, 16, 27], although the study is not designed adequately to provide strong conclusions to this effect. However, studies suggest that even in early node-negative disease, removal of at least 15 nodes remains important to improve survival [4]. Even with D1 lymphadenectomy retrieval of 15 nodes as a goal is achievable [6, 7, 9, 17]. In this study, 92% of D1 dissection cases had at least 15 nodes examined and 73% of patients with < 15 lymph nodes examined had node-negative disease. Additional analysis of T1 N0 patients (Additional file 5: Figure S4) showed no statistically significant difference in overall survival (*p* = 0.96) and disease-free survival (*p* = 0.79) between those who had < 15 and ≥ 15 lymph nodes examined. However, this may be due to the very small number of patients with < 15 nodes removed, comprising only 1.8% (33/1815) of patients with T1 N0 disease.

Worse survival outcomes were observed for patients with > 45 nodes examined when the entire patient cohort was analyzed. This may be due to the significant trend towards more nodes being removed with increasing pathologic stage and higher proportion of patients with > 45 nodes removed with advanced-stage disease. By looking at the entire patient cohort alone, it is unclear whether the poorer survival with removal of > 45 nodes is due to more advanced disease or removal of more nodes per se. Hence, analysis per stage category, was important to eliminate this concern.

This study’s findings are inconsistent with the results of a US population-based study using the Surveillance, Epidemiology, and End Results (SEER) database which showed that, for every stage, overall survival was found to be highly dependent on the number of nodes examined [5]. The different results could be explained by differences in methodology. The SEER study included low to high-volume gastric centers and excluded patients with N2–3 or T4 disease. Categories of number of removed lymph nodes and stage subgroupings were different. Additionally, 78% of patients had < 15 lymph nodes examined with a median of 8 nodes. In the present study, less than 2% of patients had < 15 nodes examined with a median of 41 nodes. Hence, in the SEER study, it is difficult to ascertain whether the survival benefit was an actual therapeutic benefit or the result of stage migration [5]. Another study using the National Cancer Database also showed positive association of OS with lymph node yield for both node-negative and node-positive disease but rate of adequate nodal retrieval was only 21–36% [22].

Distinguishing stage migration from therapeutic benefit confounds interpretation of studies demonstrating survival benefit of extended lymphadenectomy. Extensive lymphadenectomy results in higher nodal retrieval, higher probability of detecting nodal metastasis, and hence, more accurate pathologic staging [3, 4, 17–20, 27, 28, 35, 36]. Accurate staging leads to better stage-specific survival and may explain in part the benefit with D2 versus D1 dissection and better survival outcomes in Eastern versus Western centers [4, 29, 36]. Coburn et al. demonstrated poor survival for every stage with inadequate lymph node assessment [19]. Even in an Eastern high-volume center, a linear relationship between number of examine nodes and survival was demonstrated [24].

In this study, stage migration effect, while not completely eliminated, may have been reduced since most patients had D2 dissection and > 15 nodes examined. Nodal staging and stage-specific survival estimates are more accurate with at least 15 nodes examined [4, 25, 34, 35]. De Manzoni et al. suggested that D2 dissection is required for accurate gastric cancer staging given their findings that 62% of D1 dissection patients had < 15 nodes retrieved compared to 5.5% with D2 dissection [20].

Stage migration causing improved survival in other studies was suggested by positive correlation between number of examined and metastatic lymph nodes on linear regression analysis [24, 28]. Similar result was seen here but stage subgroup analysis revealed a stage-specific correlation. In stage IIIA where a survival benefit for greater nodal harvest was demonstrated, no significant correlation between number of nodal harvest and pathologic lymph nodes was found, suggesting that survival benefit in this intermediate stage may be an actual therapeutic benefit.

Nodal micrometastasis is one rationale for aggressive lymph node removal [33]. Immunohistochemical and molecular studies of resected nodes showed association of nodal micrometastasis with poor prognosis [30–32]. But, some studies on node-negative gastric cancer with 10–53% nodal micrometastasis rate failed to demonstrate survival difference between micrometastasis-positive and -negative groups [36, 37]. This may be due to the already favorable prognosis of early gastric cancer and because the micrometastasis-positive nodes were removed. The risk of nodal micrometastasis is correlated with tumor size and invasion depth and is more likely to occur in patients with lymph node metastases on conventional examination [32, 38, 39]. It is in the setting of intermediate-stage disease, therefore, where micrometastasis risk is high but systemic disease low that resection of more nodes may contribute to survival, as seen here.

A limitation in the clinical applicability of this study’s finding of improved survival with increased lymph node harvest in intermediate-stage disease is the accurate clinical identification of such patients. Preoperative and intraoperative assessment of nodal status is poorly correlated with pathologic nodal staging [40]. Hence, as mentioned in most gastric cancer guidelines, limited lymphadenectomy should be performed only in select group of T1N0 gastric cancer patients.

In our study, gastric cancer patients receiving neoadjuvant therapy were excluded from the analysis in an attempt to limit the potential confounding effect that neoadjuvant therapy may have on the ultimate number of lymph node harvested at the time of curative resection. However, there is no consensus in the literature as to whether or not the administration of neoadjuvant therapy for gastric cancer reduces the ultimate number of lymph nodes harvested at the time of curative resection [41, 42]. It is possible that our exclusion of gastric cancer patients receiving neoadjuvant therapy from our analysis could have been responsible for some portion of the survival advantage observed in intermediate stage disease patients with a greater number of lymph nodes harvested. This issue is of particular clinical interest in Western gastric cancer centers where neoadjuvant therapy is considered a standard of care for all patients except for those with very early stage disease [13, 14]. On the other hand, in Eastern gastric cancer centers, particularly in Japan and South Korea, where neoadjuvant therapy is not yet considered a standard of care, and for which only a small portion of gastric cancer patients receive neoadjuvant therapy, this issue is resultantly more difficult to assess.

This study’s strengths that allowed for good assessment of therapeutic impact of greater nodal harvest on survival and minimization of stage migration effect include the: 1) large number of cases, 2) completeness of data and follow-up, 3) performance in high-volume specialist center with standardized treatment protocols, 4) adequacy of lymph node retrieval for staging, and 5) subgroup categories according to AJCC stage. However, the limitation of the study to a single Eastern institution may preclude generalizability of results particularly in Western centers.

## Conclusion

In a high-volume gastric cancer center with standardized lymphadenectomy and retrieval of adequate number of lymph nodes for staging, removal of greater number of nodes may improve survival in patients with intermediate-stage disease.

## Additional files

Additional file 1: Figure S1. Kaplan-Meier disease-free survival curves according to total number of lymph nodes examined (≤ 30, 31-45, > 45) for all patients and for each stage subgroup. (A) All patients; (B) stage IA; (C) stage IB; (D) stage IIA; (E) stage IIB; (F) stage IIIA; (G) stage IIIB; (H) stage IIIC. (DOCX 327 kb)

Additional file 2: Figure S2. Kaplan-Meier overall survival curves according to total number of lymph nodes examined (≤ 30, 31-45, >45) for all patients and for each stage subgroup. (A) All patients; (B) stage IA; (C) stage IB; (D) stage IIA; (E) stage IIB; (F) stage IIIA; (G) stage IIIB; (H) stage IIIC. (DOCX 698 kb)

Additional file 3: Figure S3. Scatterplot and linear regression analysis of number of positive pathologic lymph nodes versus number of harvested lymph nodes for all patients and for each stage subgroup (*r* Spearman correlation value*; p* Spearman correlation test value*).* (A) All patients; (B) stage IA; (C) stage IB; (D) stage IIA; (E) stage IIB; (F) stage IIIA; (G) stage IIIB; (H) stage IIIC. (DOCX 1162 kb)

Additional file 4: Table S1. Univariate and Multivariate Analysis of Clinicopathologic Factors Associated with Overall Survival. (DOCX 42 kb)

Additional file 5: Figure S4. Kaplan-Meier overall survival curves (A) and disease-free survival curves (B) according to total number of lymph nodes examinded (< 15, ≥ 15) for T1N0 patients. (DOCX 36 kb)

## Abbreviations

AJCC: American Joint Committee on Cancer
ANOVA: Analysis of variance
ASA: American Society of Anesthesiology
BMI: Body mass index
DFS: Disease-free survival
LVI: Lymphovascular space invasion
NCC: National Cancer Center
OS: Overall survival
pN: Pathologic nodal status
PNI: Perineural invasion
pT: Pathologic tumor status
SD: Standard deviation
SEER: Surveillance, epidemiology, and end results

## References

[1] Ferlay J, Soerjomataram I, Ervik M, et al. GLOBOCAN 2012 v1.0, Cancer Incidence and Mortality Worldwide: IARC CancerBase No. 11 [Internet]. Lyon, France: International Agency for Research on Cancer; 2013. Available: http://globocan.iarc.fr. Accessed 14 Dec 2015.

[2] Sasako M (2012). Gastric cancer eastern experience. Surg Oncol Clin N Am.

[3] Coburn NG (2009). Lymph nodes and gastric cancer. J Surg Oncol.

[4] Biffi R, Botteri E, Cenciarelli S (2011). Impact on survival of the number of lymph nodes removed in patients with node-negative gastric cancer submitted to extended lymph node dissection. Eur J Surg Oncol.

[5] Smith DD, Schwarz RR, Schwarz RE (2005). Impact of Total lymph node count on staging and survival after Gastrectomy for gastric cancer: data from large US-population database. J Clin Oncol.

[6] Hartgrink HH, van de Velde CJ, Putter H (2004). Extended lymph node dissection for gastric cancer: who may benefit? Final results of the randomized Dutch gastric cancer group trial. J Clin Oncol.

[7] Cuschieri A, Weden S, Fielding J (1999). Patient survival after D1 and D2 resections for gastric cancer: long-term results of the MRC randomized surgical trial. Surgical Cooperative Group. Br J Cancer.

[8] Songun I, Putter H, Kranenbarg EMK, Sasako M, van de Velde CJ (2010). Surgical treatment of gastric cancer: 15-year follow-up results of the randomized Nationwide Dutch D1D2 trial. Lancet.

[9] Wu CW, Hsiung CA, Lo SS (2006). Nodal dissection for patients with gastric cancer: a randomised controlled trial. Lancet Oncol.

[10] Yu W, Choi G, Chung H (2006). Randomized clinical trial of Splenectomy versus Splenic preservation in patients with proximal gastric cancer. Br J Surg.

[11] Csendes A, Burdiles P, Rojas J, Braghetto I, Diaz JC, Maluenda F (2002). A prospective randomized study comparing D2 Total Gastrectomy versus D2 Total Gastrectomy plus Splenectomy in 187 patients with gastric carcinoma. Surgery.

[12] Kodera E, Fujiwara M, Ito Y, Ohashi N, Nakayama G, Koike M, Nakao A (2009). Radical surgery for gastric carcinoma: it is not an issue of whether to perform D1 or D2. Dissect as many lymph nodes as possible and you will be rewarded. Acta Chir Belg.

[13] Waddell T, Verheij M, Allum M, Cunningham D, Cervantes A, Arnold D (2014). Gastric cancer: ESMO-ESS0-ESTRO clinical practice guidelines for diagnosis, treatment, and follow-up. Eur J Surg Oncol.

[14] National Comprehensive Cancer Network (2015). NCCN Clinical Practice Guidelines in Oncology: Gastric Cancer version 3.2015 Available: http://www.nccn.org/professionals/physician_gls/pdf/gastric.pdf. Accessed 14 Dec 2015. 10.6004/jnccn.2016.0137 27697982

[15] Japanese Gastric Cancer Association (2011). Japanese gastric cancer treatment guidelines 2010 (ver.3). Gastric Cancer.

[16] Lee JH, Kim JG, Jung HK (2014). Clinical practice guidelines for gastric cancer in Korea: an evidence-based approach. J Gastric Cancer.

[17] Wagner P, Ramaswamy A, Ruschoff J, Schmitz-Moormann P, Rothmund M (1991). Lymph node counts in the upper abdomen: anatomical basis of Lymphadenectomy in gastric cancer. Br J Surg.

[18] Bunt AMG, Hermans J, van de Velde CJH, Sasako M, Hoefsloot FA, Fleuren G, Bruijn JA (1996). Lymph node retrieval in randomized trial on western-type versus Japanese-type surgery in gastric cancer. J Clin Oncol.

[19] Coburn NG, Swallow CJ, Kiss A, Law C (2006). Significant regional variation in adequacy of lymph node assessment and survival in gastric cancer. Cancer.

[20] De Manzoni G, Verlato G, Roviello F (2002). The new TNM classification of lymph node metastasis minimizes stage migration problems in gastric cancer patients. Br J Cancer.

[21] Bouvier AM, Haas O, Piard F, Roignot P, Bonithon-Kopp C, Faivre J (2002). How many nodes must be examined to accurately stage gastric carcinomas? Results from a population-based study. Cancer.

[22] Samples JE, Stitzenberg K, Meyers MO (2014). Lymph node yield and survival in gastric carcinoma. J Clin Oncol (Meet Abstr).

[23] Huang CM, Lin JX, Zheng CH, Li P, Xie JW, Lin BJ (2011). Effect of negative lymph node count on survival for gastric cancer after curative distal Gastrectomy. Eur J Surg Oncol.

[24] Kong SH, Lee HJ, Ahn HS, Kim JW, Kim WH, Lee KU, Yang HK (2012). Stage migration effect on survival in gastric cancer surgery with extended Lymphadenectomy: the reappraisal of positive lymph node ratio as a proper N-staging. Ann Surg.

[25] Karpeh MS, Leon L, Klimstra D, Brennan MF (2000). Lymph node staging in gastric cancer: is location more important than number? An analysis of 1038 patients. Ann Surg.

[26] Feinstein AR, Sosin DM, Wells CK (1985). The will Rogers phenomenon: stage migration and new diagnostic techniques as a source of misleading statistics for survival in cancer. N Engl J Med.

[27] Bunt AMG, Hogendoorn PCW, van de Velde CJH, Bruijn JA, Hermans J (1995). Lymph node staging standards in gastric cancer. J Clin Oncol.

[28] Ichikura T, Ogawa T, Chochi K, Kawabata T, Sugasawa H, Mochizuki H (2003). Minimum number of lymph nodes that should be examined for the International Union against Cancer/American joint committee on cancer TNM classification of gastric carcinoma. World J Surg.

[29] Hundahl SA, Phillips JL, Menck HR (2000). The National Cancer Data Base Report on poor survival of U.S. gastric carcinoma patients treated with Gastrectomy: fifth edition American joint committee on cancer staging, proximal disease, and the “different disease” hypothesis. Cancer.

[30] Wu ZY, Li JH, Zhan WH, He YL, Wan J (2007). Effect of lymph node micrometastases on prognosis of gastric carcinoma. World J Gastroenterol.

[31] Maehara Y, Oshiro T, Endo K (1996). Clinical significance of occult micrometastases in lymph nodes from patients with early gastric cancer who died of recurrence. Surgery.

[32] Lee E, Chae Y, Kim I, Choi J, Yeom B, Leong AS (2002). Prognostic relevance of Immunohistochemically detected lymph node micrometastasis in patients with gastric carcinoma. Cancer.

[33] Siewert JR, Kestlmeier R, Busch R (1996). Benefits of D2 lymph node dissection for patients with gastric cancer and pN0 and pN1 lymph node metastases. Br J Surg.

[34] Siewert JR, Bottcher K, Stein HJ, Roder JD (1998). Relevant prognostic factors in gastric cancer: ten-year results of the German gastric cancer study. Ann Surg.

[35] Lee HK, Yang HK, Kim WH, Lee KU, Choe KJ, Kim JP (2001). Influence of number of lymph nodes examined on staging of gastric cancer. Br J Surg.

[36] Bunt AMG, Hermans J, Smit V, van de Velde CJ, Fleuren GJ, Bruijn JA (1995). Surgical/pathologic stage migration confounds comparisons of gastric cancer survival rates between Japan and western countries. J Clin Oncol.

[37] Morgagni P, Saragoni L, Scarpi E, Zattini PS, Zaccaroni A, Morgagni D, Bazzocchi F (2003). Lymph node micrometastases in early gastric cancer and their impact on prognosis. World J Surg.

[38] Fukagawa T, Sasako M, Ito S (2010). The prognostic significance of isolated tumor cells in the lymph nodes of gastric cancer patients. Gastric Cancer.

[39] Kim JJ, Song KY, Hur H, Hur JI, Park SM, Park CH (2009). Lymph node micrometastasis in node-negative early gastric cancer. Eur J Surg Oncol.

[40] Lee SE, Ryu KW, Nam BH (2009). Prognostic significance of Intraoperatively estimated surgical stage in curatively Resected gastric cancer patients. J Am Coll Surg.

[41] Wu ZM, Teng RY, Shen JG, Xie SD, Xu CY, Wang LB (2011). Reduced lymph node harvest after neoadjuvant chemotherapy in gastric cancer. J Int Med Res.

[42] Dikken JL, van Grieken NCT, Krijnen P (2012). Preoperative chemotherapy does not influence the number of evaluable lymph nodes in resected gastric cancer. EJSO.

