# Supplementary material for: Effect of total number of harvested lymph nodes on survival outcomes after curative resection for gastric adenocarcinoma: findings from an eastern high-volume gastric cancer center
Source: BMC Cancer. 2018 Jan 12;18:73. doi: 10.1186/s12885-017-3872-6 (PMC5766983; doi:10.1186/s12885-017-3872-6)
Supplement: Supplementary file 1 — Kaplan-Meier disease-free survival curves according to total number of lymph nodes examined (≤ 30, 31-45, > 45) for all patients and for each stage subgroup. (A) All patients; (B) stage IA; (C) stage IB; (D) stage IIA; (E) stage IIB; (F) stage IIIA; (G) stage IIIB; (H) stage IIIC. (DOCX 327 kb) [file 12885_2017_3872_MOESM1_ESM.docx]

**Additional file 1: Figure S1.** Kaplan-Meier disease-free survival curves according to total number of lymph nodes examined (≤ 30, 31-45, >45) for all patients and for each stage subgroup. (A) All patients; (B) stage IA; (C) stage IB; (D) stage IIA; (E) stage IIB; (F) stage IIIA; (G) stage IIIB; (H) stage IIIC.

A

G

F

D

E

C

B

H
