# Supplementary material for: Effect of total number of harvested lymph nodes on survival outcomes after curative resection for gastric adenocarcinoma: findings from an eastern high-volume gastric cancer center
Source: BMC Cancer. 2018 Jan 12;18:73. doi: 10.1186/s12885-017-3872-6 (PMC5766983; doi:10.1186/s12885-017-3872-6)
Supplement: Supplementary file 3 — Scatterplot and linear regression analysis of number of positive pathologic lymph nodes versus number of harvested lymph nodes for all patients and for each stage subgroup (r Spearman correlation value; p Spearman correlation test value). (A) All patients; (B) stage IA; (C) stage IB; (D) stage IIA; (E) stage IIB; (F) stage IIIA; (G) stage IIIB; (H) stage IIIC. (DOCX 1162 kb) [file 12885_2017_3872_MOESM3_ESM.docx]

**Additional file 3: Figure S3.** Scatterplot and linear regression analysis of number of positive pathologic lymph nodes versus number of harvested lymph nodes for all patients and for each stage subgroup (*r* Spearman correlation value*; p* Spearman correlation test value*).* (A) All patients; (B) stage IA; (C) stage IB; (D) stage IIA; (E) stage IIB; (F) stage IIIA; (G) stage IIIB; (H) stage IIIC.

E

F

C

D

A

B

G

H
