# Supplementary material for: Effect of total number of harvested lymph nodes on survival outcomes after curative resection for gastric adenocarcinoma: findings from an eastern high-volume gastric cancer center
Source: BMC Cancer. 2018 Jan 12;18:73. doi: 10.1186/s12885-017-3872-6 (PMC5766983; doi:10.1186/s12885-017-3872-6)
Supplement: Supplementary file 4 — Univariate and Multivariate Analysis of Clinicopathologic Factors Associated with Overall Survival. (DOCX 42 kb) [file 12885_2017_3872_MOESM4_ESM.docx]

| **Additional file 4: Table S1.** Univariate and Multivariate Analysis of Clinicopathologic Factors Associated with Overall Survival | | | | | | | | | | | | | | | | | |
| --- | --- | --- | --- | --- | --- | --- | --- | --- | --- | --- | --- | --- | --- | --- | --- | --- | --- |
| Risk factor | All stages | | | | | | | |  | Stage IIIA | | | | | | | |
|  | Univariate Analysis | | | |  | Multivariate Analysis | | |  | Univariate Analysis | | | |  | Multivariate Analysis | | |
|  | HR | 95% CI | *p*^a^ | *p*^b^ |  | HR | 95% CI | *p*^a^ |  | HR | 95% CI | *p*^a^ | *p*^b^ |  | HR | 95% CI | *p*^a^ |
| Age, years |  |  |  | <.001 |  |  |  |  |  |  |  |  | <.001 |  |  |  |  |
| <65 | 1.00 |  |  |  |  | 1.00 |  |  |  | 1.00 |  |  |  |  | 1.00 |  |  |
| ≥65 | 2.27 | 2.00-2.59 | <.001 |  |  | 1.99 | 1.70-2.33 | <.001 |  | 2.23 | 1.55-3.20 | <.001 |  |  | 2.23 | 1.51-3.30 | <.001 |
| Sex^†^ |  |  |  | 0.004 |  |  |  |  |  |  |  |  | 0.256 |  |  |  |  |
| Male | 1.00 |  |  |  |  | - |  |  |  | 1.00 |  |  |  |  | - |  |  |
| Female | 0.81 | 0.70-0.93 | 0.004 |  |  | - |  |  |  | 1.25 | 0.85-1.83 | 0.256 |  |  | - |  |  |
| BMI | 0.91 | 0.89-0.93 | <.001 |  |  | 0.95 | 0.92-0.97 | <.001 |  | 1.00 | 0.94-1.06 | 0.914 |  |  | - |  |  |
| ASA |  |  |  | <.001 |  |  |  |  |  |  |  |  | 0.036 |  |  |  |  |
| I | 1.00 |  |  |  |  | 1.00 |  |  |  | 1.00 |  |  |  |  | 1.00 |  |  |
| II | 1.45 | 1.25-1.69 | <.001 |  |  | 1.25 | 1.06-1.48 | 0.008 |  | 1.39 | 0.93-2.10 | 0.111 |  |  | 1.24 | 0.82-1.89 | 0.315 |
| III | 3.19 | 2.50-4.07 | <.001 |  |  | 2.24 | 1.71-2.93 | <.001 |  | 2.40 | 1.19-4.83 | 0.014 |  |  | 1.78 | 0.84-3.44 | 0.108 |
| pT size (cm) | 1.19 | 1.17-1.21 | <.001 |  |  | 1.02 | 0.99-1.05 | 0.168 |  | 1.02 | 0.98-1.05 | 0.313 |  |  |  |  |  |
| pT category |  |  |  | <.001 |  |  |  |  |  |  |  |  | 0.191 |  |  |  |  |
| T1 | 1.00 |  |  |  |  | 1.00 |  |  |  | - |  |  |  |  | - |  |  |
| T2 | 1.78 | 1.41-2.26 | <.001 |  |  | 0.83 | 0.56-1.24 | 0.366 |  | 1.00 |  |  |  |  | - |  |  |
| T3 | 3.56 | 2.96-4.29 | <.001 |  |  | 1.10 | 0.72-1.66 | 0.663 |  | 0.82 | 0.49-1.36 | 0.445 |  |  | - |  |  |
| T4 | 9.15 | 7.72-10.85 | <.001 |  |  | 2.00 | 1.29-3.09 | 0.002 |  | 1.19 | 0.70-2.05 | 0.521 |  |  | - |  |  |
| pN category |  |  |  | <.001 |  |  |  |  |  |  |  |  | 0.191 |  |  |  |  |
| N0 | 1.00 |  |  |  |  | 1.00 |  |  |  | - |  |  |  |  | - |  |  |
| N1 | 2.23 | 1.82-2.75 | <.001 |  |  | 1.44 | 1.10-1.87 | 0.007 |  | 1.00 |  |  |  |  | - |  |  |
| N2 | 3.64 | 3.00-4.43 | <.001 |  |  | 1.79 | 1.36-2.35 | <.001 |  | 0.69 | 0.46-1.03 | 0.070 |  |  | - |  |  |
| N3 | 8.21 | 7.02-9.61 | <.001 |  |  | 3.45 | 2.64-4.50 | <.001 |  | 0.84 | 0.49-1.44 | 0.521 |  |  | - |  |  |
| Total number of LN examined |  |  |  | 0.007 |  |  |  |  |  |  |  |  | 0.047 |  |  |  |  |
| 0 – 30 | 1.00 |  |  |  |  | 1.00 |  |  |  | 1.00 |  |  |  |  | 1.00 |  |  |
| 30 – 45 | 1.11 | 0.93-1.32 | 0.240 |  |  | 1.05 | 0.86-1.28 | 0.614 |  | 1.04 | 0.65-1.66 | 0.883 |  |  | 0.87 | 0.53-1.45 | 0.580 |
| > 45 | 1.30 | 1.09-1.55 | 0.003 |  |  | 0.96 | 0.79-1.18 | 0.721 |  | 0.63 | 0.38-1.04 | 0.068 |  |  | 0.56 | 0.33-0.95 | 0.030 |
| pStage |  |  |  | <.001 |  |  |  |  |  |  |  |  |  |  |  |  |  |
| IA | 1.00 |  |  |  |  |  |  |  |  | - |  |  |  |  | - |  |  |
| IB | 1.42 | 1.07-1.89 | 0.017 |  |  |  |  |  |  | - |  |  |  |  | - |  |  |
| IIA | 1.91 | 1.45-2.52 | <.001 |  |  |  |  |  |  | - |  |  |  |  | - |  |  |
| IIB | 3.26 | 2.53-4.20 | <.001 |  |  |  |  |  |  | - |  |  |  |  | - |  |  |
| IIIA | 4.90 | 3.88-6.20 | <.001 |  |  |  |  |  |  | - |  |  |  |  | - |  |  |
| IIIB | 6.74 | 5.43-8.36 | <.001 |  |  |  |  |  |  | - |  |  |  |  | - |  |  |
| IIIC | 15.02 | 12.35-18.28 | <.001 |  |  |  |  |  |  | - |  |  |  |  | - |  |  |
| Tumor location |  |  |  | <.001 |  |  |  |  |  |  |  |  | 0.019 |  |  |  |  |
| Proximal | 1.00 |  |  |  |  | 1.00 |  |  |  | 1.00 |  |  |  |  | 1.00 |  |  |
| Middle | 0.82 | 0.68-1.00 | 0.045 |  |  | 1.27 | 1.00-1.61 | 0.051 |  | 1.16 | 0.66-2.09 | 0.607 |  |  | 0.96 | 0.52-1.80 | 0.895 |
| Distal | 0.86 | 0.72-1.03 | 0.098 |  |  | 1.35 | 1.05-1.75 | 0.020 |  | 1.95 | 1.21-3.32 | 0.010 |  |  | 1.69 | 0.99-3.02 | 0.065 |
| Whole | 3.99 | 2.93-5.44 | <.001 |  |  | 1.66 | 1.15-2.40 | 0.007 |  | 0.76 | 0.01-5.53 | 0.849 |  |  | 0.90 | 0.01-6.81 | 0.945 |
| Histologic grade* |  |  |  | <.001 |  |  |  |  |  |  |  |  | 0.392 |  |  |  |  |
| Differentiated | 1.00 |  |  |  |  | 1.00 |  |  |  | 1.00 |  |  |  |  | - |  |  |
| Undifferentiated | 1.13 | 0.98-1.29 | 0.087 |  |  | 0.85 | 0.72-1.00 | 0.045 |  | 1.00 | 0.67-1.49 | 0.999 |  |  | - |  |  |
| Others | 3.15 | 2.10-4.73 | <.001 |  |  | 2.69 | 1.74-4.15 | <.001 |  | 1.88 | 0.73-4.82 | 0.190 |  |  | - |  |  |
| Borrmann type |  |  |  | <.001 |  |  |  |  |  |  |  |  | 0.127 |  |  |  |  |
| 0 | 1.00 |  |  |  |  | 1.00 |  |  |  | 1.00 |  |  |  |  | - |  |  |
| I | 3.37 | 2.00-5.68 | <.001 |  |  | 1.73 | 0.92-3.25 | 0.089 |  | 0.54 | 0.11-1.87 | 0.392 |  |  | - |  |  |
| II | 3.50 | 2.80-4.37 | <.001 |  |  | 1.43 | 0.95-2.15 | 0.085 |  | 0.47 | 0.22-1.06 | 0.063 |  |  | - |  |  |
| III | 4.40 | 3.75-5.15 | <.001 |  |  | 1.85 | 1.28-2.68 | 0.001 |  | 0.79 | 0.44-1.60 | 0.484 |  |  | - |  |  |
| IV | 11.30 | 8.78-14.54 | <.001 |  |  | 2.27 | 1.41-3.65 | 0.001 |  | 1.80 | 0.59-4.90 | 0.270 |  |  | - |  |  |
| V | 0.90 | 0.22-3.64 | 0.886 |  |  | 1.29 | 0.31-5.42 | 0.732 |  | 0.67 | 0.01-5.21 | 0.783 |  |  | - |  |  |
| Lauren type |  |  |  | 0.508 |  |  |  |  |  |  |  |  | 0.842 |  |  |  |  |
| Intestinal | 1.00 |  |  |  |  |  |  |  |  | 1.00 |  |  |  |  | - |  |  |
| Diffuse | 1.04 | 0.90-1.19 | 0.592 |  |  |  |  |  |  | 0.89 | 0.60-1.32 | 0.570 |  |  | - |  |  |
| Mixed | 1.04 | 0.81-1.32 | 0.776 |  |  |  |  |  |  | 0.79 | 0.39-1.60 | 0.512 |  |  | - |  |  |
| Indeterminate/ Unknown | 1.27 | 0.93-1.73 | 0.132 |  |  |  |  |  |  | 1.12 | 0.53-2.35 | 0.773 |  |  | - |  |  |
| Lymphovascular invasion |  |  |  | <.001 |  |  |  |  |  |  |  |  | 0.087 |  |  |  |  |
| Absent | 1.00 |  |  |  |  | 1.00 |  |  |  | 1.00 |  |  |  |  | - |  |  |
| Present | 3.87 | 3.36-4.46 | <.001 |  |  | 1.31 | 1.08-1.60 | 0.007 |  | 1.53 | 0.94-2.47 | 0.087 |  |  | - |  |  |
| Perineural invasion^†^ |  |  |  | <.001 |  |  |  |  |  |  |  |  | 0.266 |  |  |  |  |
| Absent | 1.00 |  |  |  |  |  |  |  |  | 1.00 |  |  |  |  | - |  |  |
| Present | 3.49 | 3.03-4.01 | <.001 |  |  |  |  |  |  | 1.23 | 0.85-1.79 | 0.266 |  |  | - |  |  |
| Resection type |  |  |  | <.001 |  |  |  |  |  |  |  |  | 0.132 |  |  |  |  |
| Subtotal | 1.00 |  |  |  |  | 1.00 |  |  |  | 1.00 |  |  |  |  | - |  |  |
| Total | 1.83 | 1.59-2.11 | <.001 |  |  | 1.20 | 0.98-1.49 | 0.084 |  | 0.74 | 0.49-1.10 | 0.138 |  |  | - |  |  |
| Extended | 4.06 | 3.25-5.07 | <.001 |  |  | 1.55 | 1.16-2.06 | 0.003 |  | 0.41 | 0.13-1.31 | 0.132 |  |  | - |  |  |
| Extent of lymphadenectomy |  |  |  | 0.001 |  |  |  |  |  |  |  |  | 0.187 |  |  |  |  |
| D1 | 1.00 |  |  |  |  | 1.00 |  |  |  | 1.00 |  |  |  |  | - |  |  |
| D2 | 1.23 | 0.81-1.86 | 0.334 |  |  | 0.67 | 0.40-1.12 | 0.124 |  | 0.24 | 0.07-2.12 | 0.086 |  |  | - |  |  |
| > D2 | 2.72 | 1.52-4.88 | 0.001 |  |  | 0.73 | 0.36-1.45 | 0.362 |  | 0.16 | 0.02-1.78 | 0.081 |  |  | - |  |  |
| Adjuvant chemotherapy |  |  |  | <.001 |  |  |  |  |  |  |  |  | 0.216 |  |  |  |  |
| No | 1.00 |  |  |  |  | 1.00 |  |  |  | 1.00 |  |  |  |  | - |  |  |
| Yes | 3.05 | 2.68-3.48 | <.001 |  |  | 0.93 | 0.77-1.12 | 0.461 |  | 0.78 | 0.53-1.16 | 0.216 |  |  | - |  |  |
| Abbreviations: BMI, body mass index; ASA, American Society of Anesthesiology; pT, pathologic tumor; pN, pathologic stage  ***a*** p values indicate a relative statistical significance for each category compared to the reference category in each variable (HR 1.00)  ***b*** p values indicate statistical significance for each variable itself. Variables included the multivariate analysis were selected based on these values.  ^†^ Sex and Perineural invasion were not included in the multivariate analysis since they were not significantly associated with Total number of LN examined  * Differentiated histology included papillary adenocarcinoma, well-differentiated and moderately-differentiated tubular adenocarcinoma. Undifferentiated histology included poorly differentiated adenocarcinoma, signet ring cell adenocarcinoma, and mucinous carcinoma. Others included adenosquamous, squamous, neuroendocrine, etc. | | | | | | | | | | | | | | | | | |
