# Supplementary material for: Effect of total number of harvested lymph nodes on survival outcomes after curative resection for gastric adenocarcinoma: findings from an eastern high-volume gastric cancer center
Source: BMC Cancer. 2018 Jan 12;18:73. doi: 10.1186/s12885-017-3872-6 (PMC5766983; doi:10.1186/s12885-017-3872-6)
Supplement: Supplementary file 5 — Kaplan-Meier overall survival curves (A) and disease-free survival curves (B) according to total number of lymph nodes examinded (< 15, ≥ 15) for T1N0 patients. (DOCX 36 kb) [file 12885_2017_3872_MOESM5_ESM.docx]

P=0.96

P=0.79

Time (days) from date of surgery

Time (days) from date of surgery

A

B

**Additional file 5: Figure S4 – Supplementary.** Kaplan-Meier overall survival curves (A) and disease-free survival curves (B) according to total number of lymph nodes examinded (<15, ≥15) for T1N0 patients
